# Fat on sale: role of adipose-derived stem cells as anti-fibrosis agent in regenerative medicine

**DOI:** 10.1186/s13287-015-0246-3

**Published:** 2015-12-01

**Authors:** Manoj K. Gupta, Amrendra Kumar Ajay

**Affiliations:** Section of Islet Cell and Regenerative Biology, Joslin Diabetes Center, Harvard Medical School, Boston, MA 02215 USA; Renal Division, Department of Medicine, Brigham and Women’s Hospital, Boston, MA 02115 USA

## Abstract

The potential use of stem cells for cell-based tissue repair and regeneration offers alternative therapeutic strategies for various diseases. Adipose-derived stem cells (ADSCs) have emerged as a promising source of stem cells suitable for transplantation in regenerative medicine and wound repair. A recent publication in *Stem Cell Research & Therapy* by Zhang and colleagues reports a new finding about the anti-fibrosis role of ADSCs and conditioned media derived from them on hypertrophic scar formation in vivo.

## Commentary

Recent developments in defining the roles played by stem cells may lead to tissue repair therapies and even the eventual replacement of organs. To be used in transplantation therapy, stem cells should be easily available in abundant quantities and have the potential to differentiate into multiple lineages in a reproducible manner.

This commentary discusses the findings from Zhang and colleagues published in a recent article in *Stem Cell Research & Therapy* revealing the anti-scarring role of adipose-derived stem cells (ADSCs) and conditioned media derived from them (ADSC-CM) [1]. Wound healing is a complex process of repair and regeneration that involves the coordinated efforts of complex biological processes such as inflammation, proliferation and regeneration [2]. Stem cells, including embryonic stem cells (ESCs), induced pluripotent stem cells (iPSCs) and adult stem cells, have the capacity to proliferate and self-renew and can be differentiated into multiple lineage types [3]. Unlike ESCs derived from embryos, iPSCs are obtained from most somatic cell types after reprogramming [4]. Both of these pluripotent cell types offer enormous potential for disease modeling, drug testing and transplantation, although they are still associated with some limitations, such as immunocombatibility and teratoma formation [5].

In contrast, adult stem cells, including ADSCs, are immunocompatible and without teratogenic properties. ADSCs, multipotent stem cells, are easily derived from various adipose tissues [6]. The differentiation potential and proliferation capacity of ADSCs and soluble factors from them offer tremendous therapeutic potential for wound repair and cell-based therapy in regenerative medicine [7, 8]. The efficacy and safety of ADSCs have been determined in several preclinical and clinical studies [9]. A lot of progress has been made in characterizing and identifying specific cell-surface markers of ADSCs from subcutaneous and visceral fat depots [10, 11].

**Fig. 1:**
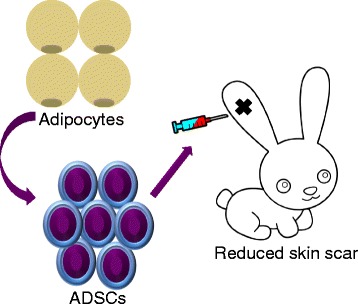
Adipose-derived stem cells (*ADSCs*) reduce hypertrophic scarring in a rabbit ear model

ADSCs are autologous, non-immunogenic, and easily available in large quantities, and seem to be a promising approach for wound repair and anti-scar therapy (Fig. 1). In a recent publication, Zhang and colleagues used ADSCs as an anti-fibrosis agent in a rabbit ear hypertrophic scarring model [1]. To this end, the authors derived ADSCs positive for CD73, CD90 and CD105 from groin fat pads of rabbit and used them to reduce scar hypertrophy in the ear scarring model in rabbit. Using ultrasonography and hematoxylin and eosin staining, they found that the scar elevation index was significantly decreased in scars treated with ADSCs and ADSC-CM. Also, collagen fibers were regularly arranged in the ADSC-treated groups compared with control groups. These findings were confirmed by real time PCR—lower expression of collagen type 1 and alpha smooth muscle actin in ADSC- and ADSC-CM-treated scars—proving that these adult stem cells have anti-fibrosis characteristics. In this elegant study, the authors observed a large number of DiI-labeled ADSCs in the scar tissue even after 3 weeks of initial treatment, indicating the active involvement of ADSCs in wound repair. However, they were not able to determine the survival rate of the ADSCs due to only temporary labeling with the dye. Therefore, lineage tracing until the end-point will be essential in any such future studies, which is the only way to discriminate between tissue regeneration in situ and stem cell-based wound healing.

Zhang and colleagues’ study not only characterized the ADSCs by surface markers, but also demonstrated the successful trans-differentiation of ADSCs into adipocytes and osteocytes, confirmed by using oil red O staining and alizarin red S. Their work is also well supported by a similar study in which the authors demonstrated that bone marrow-derived mesenchymal stem cells played important roles in wound repair and tissue remodeling dependent on p53 using the same hypertrophic scarring model in rabbit [12].

## Conclusion

Zhang and colleagues’ study is impressive, showing the anti-scarring effect of ADSCs and raising several questions for future investigations (Fig. 1). What are the key transcriptional factors and molecular pathways initially involved in lineage-specific differentiation of ADSCs? What role do these cells have as precursors of various somatic cell types, including fibroblasts and endothelial cells? Are ADSCs safe to use as an anti-fibrosis agent? What is the survival rate of these stem cells during transplantation? And, most importantly, how dependent is ADSC differentiation potential on their site of origin and the donor’s age and gender? These are a few of the myriad questions remaining to be answered regarding the use of ADSCs in wound repair and transplantation therapies. Answers to these questions may help to define strategies for the treatment of wounds in patients with various disease backgrounds, such as diabetes, scleroderma, burns, and epidermolysis bullosa hereditaria.

## Abbreviations

ADSC: Adipose-derived stem cell
ADSC-CM: ADSC-derived conditioned media
ESC: Embryonic stem cell
iPSC: Induced pluripotent stem cell
PCR: Polymerase chain reaction
